# Magneto-Absorption Spectra of Laser-Dressed Coupled Quantum Dot–Double Quantum Ring

**DOI:** 10.3390/nano15110869

**Published:** 2025-06-05

**Authors:** Doina Bejan, Cristina Stan, Alina Petrescu-Niță

**Affiliations:** 1 Faculty of Physics, University of Bucharest, 030018 Bucharest, Romania; doinita.bejan@unibuc.ro; 2 Faculty of Applied Sciences, National University of Science and Technology POLITEHNICA, 060042 Bucharest, Romania; alina.petrescu@upb.ro

**Keywords:** dot–double quantum rings, magnetic field, Aharonov–Bohm oscillations, laser field, nonlinear optical absorption

## Abstract

We investigate 3D quantum dot–double quantum ring structures of GaAs/Al_0.3_Ga_0.7_As submitted to the combined action of a non-resonant intense laser and an axial magnetic field. We study three representative geometries with the dot height larger, comparable or lower than the ring height. The intense laser field can change the confinement potential of the dot–double ring into dot–triple-ring or –multiple-ring potentials. Also, depending on the dot height, it increases/decreases the absorption of the structure. Under magnetic field, the energy spectra display Aharonov–Bohm oscillations characteristic of a single effective ring covering almost both rings, with a period controlled by the dot height. For large and medium dot height, the magnetic field lowers the absorption and leads to splitting and/or the apparition of two peaks, one that goes to red and the other to blue. In the presence of both fields, the spectra show different characteristics. The dot height and the external fields are thus proved to be efficient tools in controlling the absorption spectra, a useful feature in designing dot–double ring-based devices.

## 1. Introduction

Semiconductor quantum dots and rings stand among the most used nanostructures for dedicated technological applications in a variety of fields. Exploiting their distinctive optical and quantum properties, such as the phase-coherent transport of electrons, the selective manipulation of electronic states, and unique spectroscopic signatures, important applications were reported for optical and sensing detection [1], photodetectors [2,3], light-emitting diodes [4,5,6], quantum computing and information processing [7,8], spintronics [9,10], bioimaging and medicine [11,12].

In searching for new types of materials, different combinations of semiconductor-based double or triple rings or combinations of dots and rings are important candidates integrating the advantages of both types of nanostructures in a synergetic manner. There are systematical studies related to the properties of quantum rings laterally or vertically coupled [13,14,15,16,17,18], concentric double rings [19,20,21,22,23,24,25] or triple rings [26,27,28,29,30] under external fields. Also, the combination of quantum dot with quantum ring into a single, multi-functional complex has been intensively investigated both experimentally [31,32,33] and theoretically [34,35,36,37,38].

The electro-optical properties of quantum dot and ring semiconductor nanostructures are significantly changed by external magnetic fields. Depending on the magnitude and orientation of the field, the lifting of the degeneracy of the energy levels, anti-crossings, the creation of new hybrid states and specific phenomena such as the Aharonov–Bohm effect with distinctive interference patterns were observed with or without the presence of spin–orbit coupling [39,40,41,42,43,44,45,46].

In recent decades, attention is increasingly focused on the tailoring of the electronic and optical properties of low-dimensional semiconductor nanostructures using high-frequency laser fields [47,48,49,50,51]. It was also demonstrated by Chakraborty [52] how, with the help of an intense laser field, it is possible to restore the isotropic physical properties in anisotropic quantum rings. The external laser produces a controllable deformation of the confining potential without any change in the physical structure, allowing for the controllable tuning of energy spectra and electron localization. Using a 3D finite element model for the laser dressing of the confining potential, Radu et al. [53] analyzed important nonlinear optical effects of the electronic states induced in the energy spectra by manipulating the laser-dressing parameter. Theoretical studies on the simultaneous effects of magnetic and laser fields can be found in [47,50,51,54].

Studies on different configurations and geometries of nanostructures are still highly active topics of investigation required by the practical need of an optimal enhancement of the quantum confinement that allows a precise engineering of the energy and localization of the electronic states. The quantum dot–double ring (QDDR) structure is a good example. A large QDDR of about 360 nm was realized through droplet epitaxy by Somaschini et al. [31] but it gives low responses to laser field. In a previous paper [55], we have already studied the effects of geometry variation on the optoelectronic properties of a smaller similar structure.

In the present paper, we demonstrate that small QDDR structures are highly responsive to non-resonant intense laser and magnetic fields, thus being more interesting for potential applications. Using 3D numerical analysis, the energy spectra and magneto-absorption response are systematically discussed in close relation with the changes induced in wave function localization.

The paper is organized as follows. In Section 2, we describe the theoretical framework for QDDR. The electronic and optical properties are presented and analyzed comparatively in Section 3. Finally, the conclusions are summarized in Section 4.

## 2. Theory

We consider the electrons confined in the coupled quantum dot–double quantum ring made of GaAs embedded in an Al_0.3_Ga_0.7_As matrix. The structure is submitted to an intense laser field non-resonant with the structure acting in the *x–y* plane or/and to a magnetic field oriented along the *z*-axis.

In the absence of the external fields, the Schrödinger equation for the electron in this structure is:

(1) $$\left(-\frac{\hbar^{2}}{2}\nabla \left(\frac{1}{m^{*}\left(x,y\right)}\nabla \right)+V_{c}\left(x,y,z\right)\right)\psi \left(x,y,z\right)=E\psi \left(x,y,z\right).$$

The electron confining potential $V_{c}\left(\vec{r}\right)$ and the effective mass of the electron $m^{*}\left(x,y\right)$ of the axial symmetric structure can be written using the Heaviside step function *H* as follows:

(2) $$\begin{array}{l} V_{c}\left(\vec{r}\right)=V_{0}\left(H\left(-z\right)+H\left(z-h\left(\rho \right)\right)\right)\\ m^{*}\left(x,y\right)={m^{*}}_{GaAs}+\left({m^{*}}_{AlGaAs}-{m^{*}}_{GaAs}\right)\left(H\left(-z\right)+H\left(z-h\left(\rho \right)\right)\right) \end{array}$$

where $\rho =\sqrt{x^{2}+y^{2}}$, $V_{0}$ is the barrier potential for electrons in GaAs/Ga_0.7_Al_0.3_As and $h\left(\rho \right)$ is the height profile constructed as a superposition of three Gaussian functions as described in [55]. The QDDR structures are built by revolving the height profile around the *z*-axis.

The non-resonant, intense laser field (ILF) is a linearly polarized monochromatic radiation described by the potential vector of amplitude *A*_0_*_L_* and angular frequency $\omega_{L}$:

(3) $${\vec{A}}_{L}\left(t\right)=\hat{x}A_{0L}cos\left(\omega_{L}t\right)$$

The static axial magnetic field $\vec{B}=B\hat{z}$ can be considered as derived from the potential vector ${\vec{A}}_{m}=B/2\left(-y,x,0\right)$, $\vec{B}=\nabla \times {\vec{A}}_{m}$. Here, $\hat{x}$ and $\hat{z}$ are the unit vectors along the *x* and *z* axes, respectively.

In the presence of both fields, the electron motion is described by the solution of the time-dependent Schrödinger equation:

(4) $$\left(\left(\vec{p}+e{\vec{A}}_{L}\left(t\right)+e{\vec{A}}_{m}\right)\frac{1}{2m^{*}\left(x,y\right)}\left(\vec{p}+e{\vec{A}}_{L}\left(t\right)+e{\vec{A}}_{m}\right)+V_{c}\left(\vec{r}\right)\right)\Psi \left(x,y,z,t\right)=i\hbar \frac{\partial \Psi \left(x,y,z,t\right)}{\partial t}$$

Performing the Kramers–Henneberger unitary transformation [47], Equation (4) can be written as a time-independent equation:

(5) $$\left(-\frac{\hbar^{2}}{2}\nabla \left(\frac{1}{m^{*}\left(x,y\right)}\nabla \right)+V_{d}\left(x,y,z,\alpha_{0}\right)+U_{md}\left(x,y,\alpha_{0}\right)\right)\Phi \left(x,y,z\right)=E\Phi \left(x,y,z\right)$$

where

(6) $$V_{d}\left(x,y,z,\alpha_{0}\right)=\frac{\omega_{L}}{2\pi }\int_{0}^{2\pi /\omega_{L}}V_{c}\left(x+\alpha_{0}\cos\left(\omega_{L}t\right),y,z\right)dt$$

and

(7) $$\begin{array}{cc} U_{md}\;\left(x,y,\alpha_{0}\right) & =\frac{\omega_{L}}{2\pi }\int_{0}^{2\pi /\omega_{L}}\left(\frac{e^{2}B^{2}}{8m^{*}}\left({\left(x+\alpha_{0}\cos\left(\omega_{L}t\right)\right)}^{2}+y^{2}\right)+\frac{ieB}{2m^{*}}\left(y\frac{\partial }{\partial x}-\left(x+\alpha_{0}\cos\left(\omega_{L}t\right)\right)\frac{\partial }{\partial y}\right)\right)dt\\ & =\frac{e^{2}B^{2}}{8m^{*}\left(x,y\right)}\left(x^{2}+\frac{\alpha_{0}^{2}}{2}+y^{2}\right)+\frac{ieB}{2m^{*}\left(x,y\right)}\left(y\frac{\partial }{\partial x}-x\frac{\partial }{\partial y}\right) \end{array}$$

The value $\alpha_{0}$ is the laser-dressing parameter defined as [56]:

(8) $$\alpha_{0}\left(x,y\right)=\frac{eA_{0L}}{m^{*}\left(x,y\right)\omega_{L}}=\frac{e}{m^{*}\left(x,y\right)\omega_{L}^{2}}\sqrt{\frac{2I_{L}}{c\varepsilon_{0}}}$$

Here, *I_L_* is the ILF irradiation, $\varepsilon_{0}$ is the vacuum dielectric permittivity and *c* the vacuum speed of light. Therefore, the laser parameter $\alpha_{0}$ is a measure of ILF irradiation and is position-dependent through the effective mass. We mention that the lower limit for the application of the dressing model for quantum structures is the Terahertz frequency [30].

The energy eigenvalues *E* and eigenfunctions $\Phi \left(x,y,z\right)$ were calculated numerically using FEM (Finite Element Method) as incorporated by COMSOL Multiphysics^®^ software (v.5.6) [57]. The spatial domain of integration of the model has a cylindrical shape, coaxial with the QDDR, with a radius and a height twice the corresponding dimensions of the structure. We used an adaptative, free tetrahedral-type mesh and Dirichlet conditions for the boundary of the cylindrical domain.

Under the action of a probe laser of variable angular frequency ω, the absorption spectra of the QDDR system can be recorded. Within the compact density-matrix formalism under the steady state conditions, the nonlinear absorption coefficient for intra-band transitions starting from the ground state can be written as [58]:

(9) $$\alpha_{1j}\left(\omega \right)=\frac{\omega N\mu_{1j}^{2}T_{2}}{\varepsilon_{0}\hbar cn_{r}}\frac{\left|J_{0}^{2}\left(\frac{\left|\mu_{\mathrm{jj}}-\mu_{11}\right|E_{0}}{\hbar \omega }\right)-J_{2}^{2}\left(\frac{\left|\mu_{\mathrm{jj}}-\mu_{11}\right|E_{0}}{\hbar \omega }\right)\right|}{1+T_{2}^{2}{\left(\omega -\omega_{j1}\right)}^{2}+{\bar{\mu }}_{1j}^{2}E_{0}^{2}T_{1}T_{2}/\hbar^{2}},$$

where

(10) $${\bar{\mu }}_{1j}=\mu_{1j}\left(J_{0}\left(\frac{\left|\mu_{\mathrm{jj}}-\mu_{11}\right|E_{0}}{\hbar \omega }\right)+J_{2}\left(\frac{\left|\mu_{\mathrm{jj}}-\mu_{11}\right|E_{0}}{\hbar \omega }\right)\right).$$

In Equations (9) and (10), $J_{0},J_{2}$ are the first-kind Bessel functions, *N* is the electron density, *T*_1_ is the population decay time, *T*_2_ is the dephasing time and *n_r_* is the refractive index. $E_{0}$ is the amplitude of the probe laser electric field and $\omega_{j1}=\left(E_{j}-E_{1}\right)/\hbar$. The dipole moment matrix elements $\mu_{ij}$, defined as:

(11) $$\mu_{ij}=e\langle \Phi_{i}\left(x,y,z\right)|x|\Phi_{j}\left(x,y,z\right)\rangle ,$$

are calculated for a probe laser polarized along the *x*-axis acting on the dot–double ring.

## 3. Results

**Figure 1:**
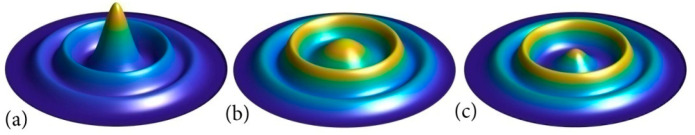
The QDDR structure in the absence of the external fields for (**a**) *h_d_* = 20 nm; *w_d_* = 5 nm; (**b**) *h_d_* = 8 nm; *w_d_* = 8 nm; (**c**) *h_d_* = 6 nm; *w_d_* = 5 nm.

In the following paragraphs, we present the results concerning the effects of the ILF and static magnetic field on the electronic and optical properties of the dot–double quantum ring structure for the three distinct geometries illustrated in Figure 1.

The height and width of the dot are considered as variable parameters in the following because in our previous paper [55], they had the strongest influence on the absorption spectra. For the three representative geometries chosen here, we used the following values for the two rings: $h_{1}$ = 7 nm; $w_{1}$ = 2.5 nm; $\rho_{1}$ = 14 nm for the inner ring and $h_{2}$ = 3.5 nm; $w_{2}$ = 2.8 nm; $\rho_{2}$ = 21 nm for the outer ring. In the first case (Figure 1a), the dot is considerably higher than the first ring ($h_{d}$ = 20 nm; $w_{d}$ = 5 nm); in the second case (Figure 1b), the dot and inner ring have comparable heights ($h_{d}$ = 8 nm; $w_{d}$ = 8 nm); and in the third case (Figure 1c), the dot is shorter than the inner ring ($h_{d}$ = 6 nm; $w_{d}$ = 5 nm).

Other parameters used in our calculations are as follows: $m{*}_{GaAs}=0.067\cdot m_{0}$; $m{*}_{GaAlAs}=0.093\cdot m_{0}$ (where $m_{0}$ is the mass of a free electron); $V_{0}$ = 262 meV [25]; $n_{r}=3.55$; *T*_1_ = 10 ps; *T*_2_ = 5 ps [58]; $N=5\times 10^{22}\;m^{-3}$.

### 3.1. Electronic Properties of Quantum Dot–Double Quantum Ring in Laser and Magnetic Fields

**Figure 2:**
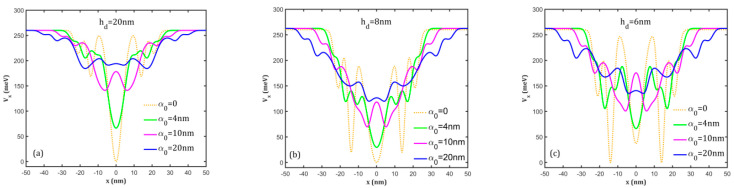
The laser-dressed potential profiles on the *x*-direction at different values of the laser parameter α_0_ for (**a**) *h_d_* = 20 nm; (**b**) *h_d_* = 8 nm; (**c**) *h_d_* = 6 nm.

In Figure 2, we present the effect of the intense *x*-polarized laser field on the potential profile. It clearly destroys the cylindrical symmetry of the rings deforming the dressed potential along the *x*-axis. For the three geometries considered here, several effects can be noticed: (i) the bottoms of the potentials corresponding to the dot and rings are raised and a dot–triple ring structure appears; this is more visible for the geometry with a low dot height (Figure 2c); (ii) the widths of upper parts of the potentials increase proportionally to the laser parameter $\alpha_{0}$; (iii) for $\alpha_{0}$ = 10 nm, the dot potential is separated into two wells, so the potential now corresponds to a triple ring structure for all geometries; (iv) for $\alpha_{0}$ = 20 nm, the dot potential becomes very small but it is still separated into two wells; the potentials of the two rings collapse in a single ring potential, but two new wells appear on the upper side, so the whole potential now describes a four-ring structure (this feature is more obvious in Figure 2c). Therefore, the confinement potential of QDDR can be continuously modified by the THz laser field that determines transitions from a dot–double ring structure to dot–triple-ring or –multiple-ring structures for a single material sample.

**Figure 3:**
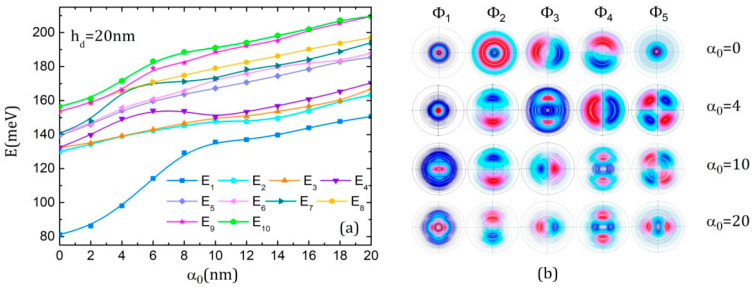

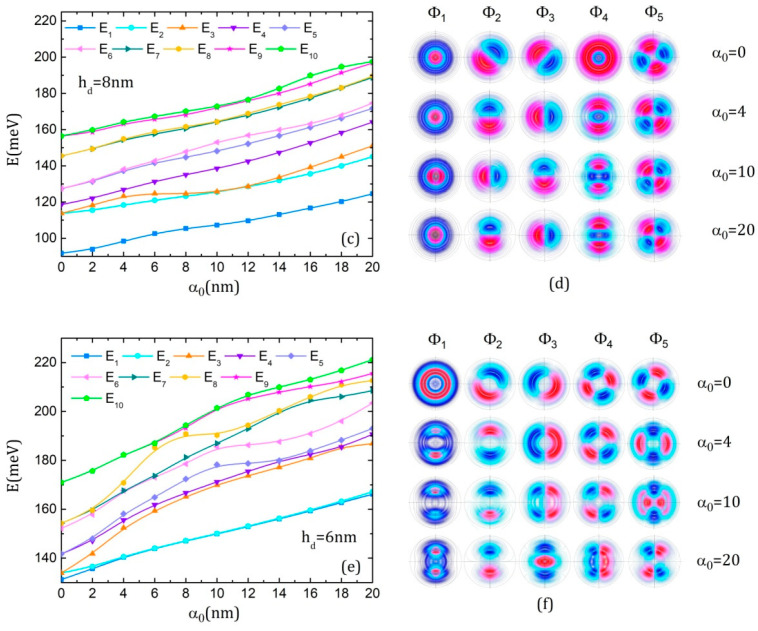
The energies of the ten lowest states of the electron in QDDR as functions of the laser parameter α_0_ and the *x-y* projections of the first five WFs represented using 300 contours lines at different values of α_0_, in the absence of the magnetic field: (**a**,**b**) *h_d_* = 20 nm; (**c**,**d**) *h_d_* = 8 nm; (**e**,**f**) *h_d_* = 6 nm.

Figure 3 illustrates the effects of the ILF on the energies and the associated wave functions of the QDDR for the three considered geometries. For ease of understanding, we comment on them together. As a general observation, all energies increase because of the potential rise on the *x*-axis with the laser parameter. The ground state is a single state for all considered values of the laser parameter for $h_{d}$ = 20 nm and $h_{d}$ = 8 nm. However, because of the presence of the dot, not all the excited state energies come into pairs as it happens for a multiple-ring structure. There are several single states besides the ground state for each structure. For instance, it can be seen in the first rows of Figure 3b,d,f that in the absence of ILF ($\alpha_{0}$ = 0), for $h_{d}$ = 20 nm, Φ_1_, Φ_2_ and Φ_5_ (also Φ_10_ represented in [55], Figure 3a, last row) have an axial symmetry, being s-like states with the quantum magnetic number *m =* 0 [51]. Moreover, Φ_1_ and Φ_5_ are located on the dot while Φ_2_ is located on the whole structure. In the other cases, Φ_1_ and Φ_4_ for $h_{d}$ = 8 nm or Φ_1_ and Φ_6_ (represented in [55], Figure 3a, second row) for $h_{d}$ = 6 nm are s-like states.

At the increment of ILF intensity, the structural change in the QDDR potential is strongly reflected in the ground state wave functions (WFs). For instance, for $h_{d}$ = 20 nm (Figure 3b, first column), at low values of $\alpha_{0}$, the WF fills the dot continuously. At $\alpha_{0}$ = 10 nm, as the potential of the dot is split in two wells, the WF is split also in two lobes and no longer fills the central part of the dot. At $\alpha_{0}$ = 20 nm, the WF begins to refill the dot as the splitting in the dot potential becomes smaller. The extension of the ground state WF into the inner ring region at $\alpha_{0}$ increment, due to the rise in the potential width, is also obvious. Similar effects can be noticed for the ground state WF for $h_{d}$ = 8 nm (Figure 3d, first column). For $h_{d}$ = 6 nm (Figure 3f, first column), it is interesting to notice that for low $\alpha_{0}$ values, the ground state WF covers only the rings because their potentials are lower than of the central dot. At higher $\alpha_{0}$ values, the ground state WF is split into two lobes that begin to cover the central dot and are restrained into the dot and first ring where the potential takes the lowest values.

The excited levels are also affected by the rise in ILF intensity. The initial degeneracy of the excited levels with quantum magnetic numbers *m*, *−m* [51,55,59] is raised and new pairs of degenerate levels are formed, thus generating a structure with multiple anti-crossings in the conduction band. At each anti-crossing, the wave functions of the involved levels change their symmetry. For instance, for $h_{d}$ = 20 nm, in the absence of ILF, (E_3_, E_4_) form a degenerate pair that separates with ILF strengthening, and at $\alpha_{0}$ = 4 nm, E_3_ and E_2_ have an anti-crossing that leads to interchanging their WFs—as seen in Figure 3b, second row. Another anti-crossing is produced at $\alpha_{0}$ = 10 nm between E_3_ and E_4_, so their WFs are also interchanged (Figure 3b, third row). For $h_{d}$ = 8 nm, (E_2_, E_3_), which are degenerated initially, separate and show an anti-crossing at $\alpha_{0}$ = 10 nm that exchanges their WFs (Figure 3d, third row). For $h_{d}$ = 6 nm, a similar observation can be made for the pairs (E_2_, E_3_) and (E_4_, E_5_). However, starting with $\alpha_{0}$ = 4 nm, (E_1_, E_2_) become degenerated (Figure 3f, first and second rows).

**Figure 4:**
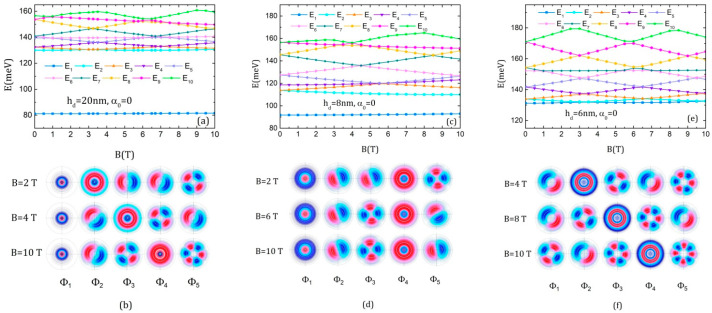
The energies of the ten lowest states of the electron in QDDR as functions of the magnetic field B and the *x-y* projections of the first five WFs represented using 300 contours lines at different values of B, in the absence of the intense laser: (**a**,**b**) *h_d_* = 20 nm; (**c**,**d**) *h_d_* = 8 nm; (**e**,**f**) *h_d_* = 6 nm.

Figure 4 presents the energy spectra and the associated wave functions of the three QDDR structures in magnetic field without ILF. By comparative observations, it is clear that the dot height strongly influences the spectra. For instance, for the QDDR with $h_{d}$ = 20 nm and $h_{d}$ = 8 nm, the ground state energy increases very slightly with the magnetic field and is well separated from the excited states as its WF is localized mainly on the dot. The excited state energies show Aharonov–Bohm oscillations (Figure 4a,c). On the contrary, for the QDDR with $h_{d}$ = 6 nm, the ground level is not separated from the excited states as their WFs are localized only on the rings, and therefore the whole energy spectrum presents Aharonov–Bohm oscillations (Figure 4e). We mention that the observed oscillations are characteristic of a single large effective ring with a period of oscillation dependent on the dot height. As the WFs show, this effective ring covers both ring regions for the excited states of QDDR with $h_{d}$ = 20 nm, 6 nm, and the inner ring and partially the outer one for $h_{d}$ = 8 nm. This feature is a consequence of the fact that the inner and outer rings are close to each other, so the WFs are generally localized in both rings for $h_{d}$ = 20 nm, 6 nm, or in the inner ring and partially in the outer ring for $h_{d}$ = 8 nm. In this latter case, the medium radius of the effective ring is lower, so the period of the oscillations is larger since it is inversely proportional to the medium radius of the ring (a demonstration can be found in [59]).

The presence of the s-like states among the excited states leads to the apparition of slowly increasing energy levels that do not modify the Aharonov–Bohm oscillations, being superposed on them. For instance, in the energy spectrum of QDDR with $h_{d}$ = 20 nm, E_2_ and E_6_ behave like E_1_, increasing very slightly with B. Levels E_2_ and E_3_ cross at B = 3.5 T, exchanging their WFs (Figure 4b, first and second row), so that Φ_2_ becomes a p-like state that keeps this feature up to B = 10 T while Φ_3_ becomes an s-like state. After (E_3_, E_4_) crossing at B = 7 T, Φ_4_ becomes a single state and Φ_3_ obtains d-like symmetry (Figure 4b, last row). Also, due to the successive crossing of E_5_ with E_6_ and E_4_, Φ_5_ acquires d-like character with *m* = ±2 and four antinodes after 3.5 T, but it becomes f-like with *m* = ±3 and six antinodes after 7 T.

In the energy spectrum of QDDR with $h_{d}$ = 8 nm, Φ_4_ is a single state for all B values. The triple crossing at B = 5.5 T determines the exchange of Φ_3_ and Φ_5_ without altering Φ_4_ (Figure 4d).

For QDDR with $h_{d}$ = 6 nm, E_1_ and E_2_ cross at 3.5 T, exchanging their WFs (Figure 4f, first row). The crossing between E_2_ and E_3_ at 6.5 T determines Φ_3_ to be s-like and Φ_3_ d-like (Figure 4f, second row). Finally, after the crossing of (E_1_, E_2_) and (E_3_, E_4_) at 9.5 T, Φ_1_ behaves d-like and Φ_4_ s-like (Figure 4f, last row).

**Figure 5:**
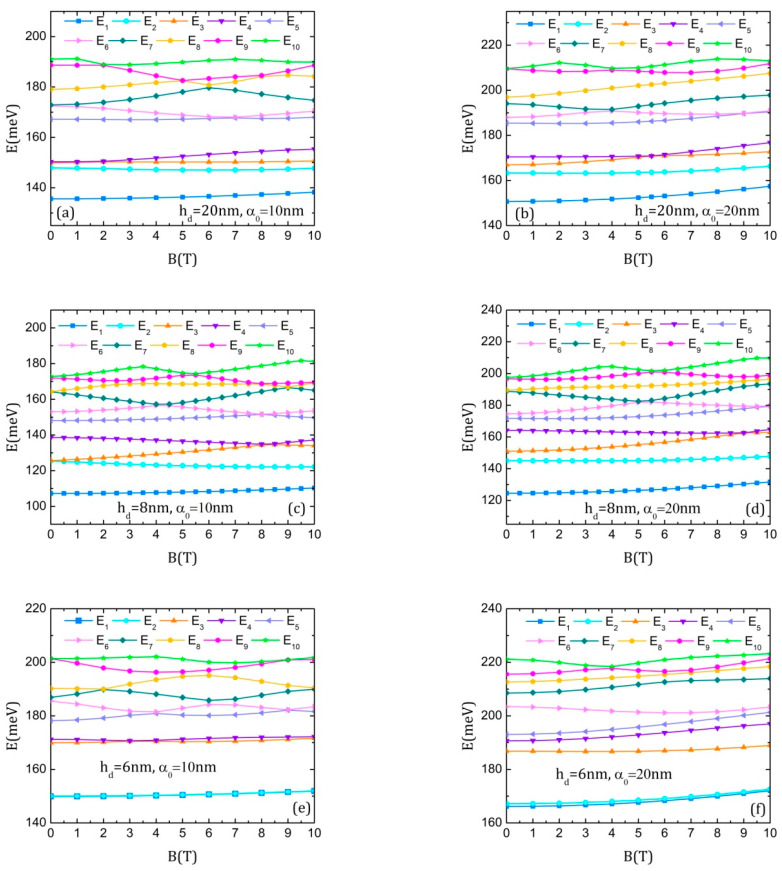
The energies of the ten lowest states of the electron in DDQR as functions of the magnetic field B, in the presence of the intense laser: (**a**) *h_d_* = 20 nm, α_0_ = 10 nm; (**b**) *h_d_* = 20 nm, α_0_ = 20 nm; (**c**) *h_d_* = 8 nm, α_0_ = 10 nm; (**d**) *h_d_* = 8 nm, α_0_ = 20 nm; (**e**) *h_d_* = 6 nm, α_0_ = 10 nm; (**f**) *h_d_* = 6 nm, α_0_ = 20 nm.

We are particularly interested in observing the modification induced in the conduction band by the simultaneous action of both ILF and magnetic field. After some trials, we found that the most important changes are those induced by the magnetic field for a given laser parameter. Consequently, we investigated the energy spectra obtained for rather large values of $\alpha_{0}$, namely, $\alpha_{0}$ = 10 nm and 20 nm, presented in Figure 5 for the three considered geometries. Some general features can be noticed: (i) the Aharonov–Bohm oscillations were washed out because the linear polarized ILF leads to the loss of azimuthal symmetry, so one can no longer assign a quantum magnetic number to the states; (ii) some oscillations, not very clearly defined, appear for the upper levels; (iii) the spectra present only anti-crossings accompanied by a change in the symmetry of the involved wave functions (largely discussed and illustrated in previous cases); (iv) the increment in ILF intensity pushes the whole spectrum to higher energy and removes the degeneracy of the levels (only the upper levels are still degenerated for $h_{d}$ = 20 nm and $h_{d}$ = 8 nm); (v) the ground levels are more sensitive to B for larger $\alpha_{0}$, showing a parabolic increment; (vi) the variation in the energies with B for the QDDR with $h_{d}$ = 6 nm is very different, the ground level and first excited state being now degenerated and separated from the rest of the spectrum. All these features will strongly influence the absorption spectra.

### 3.2. Optical Properties of Quantum Dot–Double Quantum Ring in Laser and Magnetic Fields

To properly understand the nonlinear optical absorption spectra of QDDR submitted to non-resonant laser and magnetic fields, we discuss them in connection with the dimensionless oscillator strength defined as [60]:

(12) $$O_{1-j}=\frac{2{m^{*}}_{GaAs}}{e^{2}\hbar^{2}}\left(E_{j}-E_{1}\right)\mu_{1j}^{2}.$$

The values of thus defined oscillator strength range from 0 to 1. High values indicate strong transitions, while low values indicate weak transitions. In the following, we only consider the transitions from the ground state to the lowest four excited states, so each absorption coefficient is the sum over $\alpha_{1j}$, *j* = 2–5.

**Figure 6:**
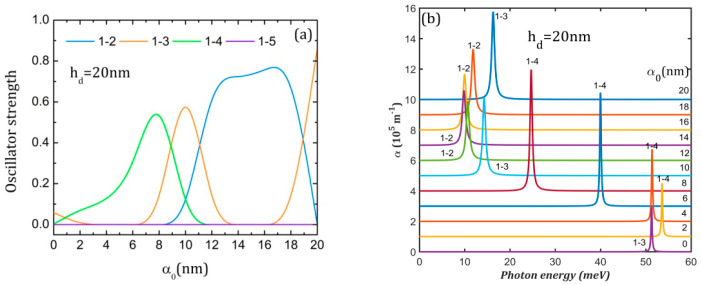

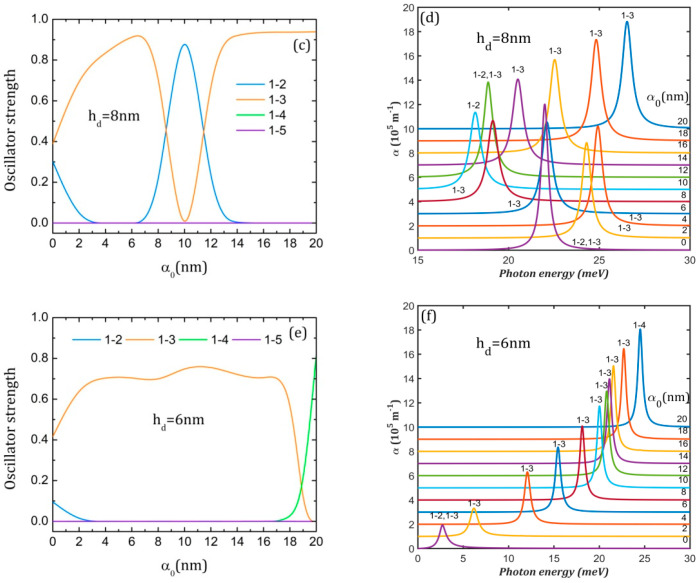
The oscillator strength (**a**,**c**,**e**) and the absorption spectra (**b**,**d**,**f**) at different values of the laser parameter α_0_ for the three QDDR structures considered. The transitions are indexed as 1–j, from the ground level to the excited j level. To avoid spectra overlapping, each spectrum is translated on the vertical axis.

Figure 6a,c,e present the oscillator strength behavior with the increment in the laser parameter. Since the ground state is always symmetric relative to the *x* and *y* axes, the *x*-polarized probe laser can only determine transitions to states that are either anti-symmetric or do not have a specific symmetry relative to the *y*-axis. In all graphs, *O*_1–5_ is zero because Φ_5_ is either symmetric or has a node on the *x*-axis (see Figure 3b,d,f, last column), so the transition 1–5 is forbidden.

For $h_{d}$ = 20 nm, we observe in Figure 6a significant values for *O*_1–3_, *O*_1–4_ and *O*_1–2_. As can be inferred from Figure 3b, at $\alpha_{0}$ = 0, Φ_3_ does not have a specific symmetry relative to the *y*-axis implying *O*_1–3_ ≠ 0, but Φ_4_ takes very low values on *x*-axis, so *O*_1–4_ is negligibly small. At $\alpha_{0}$ = 4 nm, Φ_3_ is symmetric and Φ_4_ is anti-symmetric to the *y*-axis, resulting in *O*_1–3_ = 0 and *O*_1–4_ ≠ 0, while at $\alpha_{0}$ = 10, 20 nm, the reverse is true. For $h_{d}$ = 8 nm, *O*_1–2_ and *O*_1–3_ show an oscillatory behavior (Figure 6c). At $\alpha_{0}$ = 0, Φ_2_ and Φ_3_ do not have a specific symmetry relative to the *y*-axis, and *O*_1–2_*, O*_1–3_ ≠ 0 (Figure 3d). At $\alpha_{0}$ = 4, 20 nm, Φ_2_ has a node on the *x*-axis while Φ_3_ is anti-symmetric, implying that *O*_1–2_ = 0, *O*_1–3_ ≠ 0, while at $\alpha_{0}$ = 10 nm, the reverse is true. For $h_{d}$ = 6 nm, *O*_1–3_ takes large values for all laser parameter values, excepting $\alpha_{0}$ = 20 nm, where Φ_3_ becomes symmetric to the *y*-axis, so *O*_1–3_ = 0 (Figure 3f and Figure 6e). *O*_1–4_ takes a large value only at $\alpha_{0}$ = 20 nm because Φ_4_ becomes anti-symmetric to the *y*-axis.

The absorption spectra represented in Figure 6 are calculated for a probe laser with an irradiation of 10^7^ W/m^2^. It can be noticed that the QDDR spectra consist of a single peak for all cases.

For $h_{d}$ = 20 nm (Figure 6b), the absorption peak corresponds to a 1→3 transition in the absence of the laser field. At the increment of the laser parameter, it is replaced by a 1→4 peak that goes to lower energies up to $\alpha_{0}$ = 10 nm because the ground state energy increases faster than for excited states. The single peak of these spectra is red-shifted from 53.58 meV to 9.82 meV for $\alpha_{0}$ = 14 nm and then blue-shifted up to 19.78 meV. The largest absorption maximum of 7.96 × 10^5^ m^−1^ is obtained for $\alpha_{0}$ = 8 nm. The presence of ILF increases the absorption of the nanostructure since, in its absence, the absorption is only 2.94 × 10^5^ m^−1^.

For $h_{d}$ = 8 nm, the interplay and oscillatory behavior in energy of 1→2 and 1→3 peaks that are restricted to a limited region of energies (between 20 and 40 meV) can be seen in Figure 6d. This is correlated with the similar increment in the energies of the ground and excited states. Even if the peaks are more intense than for the QDDR with $h_{d}$ = 20 nm, the intense laser field lowers nanostructure absorption since the largest absorption maximum of 12.02 × 10^5^ m^−1^ is obtained for $\alpha_{0}$ = 0 due to the superposition of 1→2 and 1→3 peaks. The minimum absorption of 6.66·10^5^ m^−1^ is obtained for $\alpha_{0}$ = 10 nm.

For $h_{d}$ = 6 nm (Figure 6f), at the increment of the laser parameter, a blue-shift can be observed in the peaks due to the increased separation between the ground state and the excited states and the rise in peak intensity. For instance, in the absence of ILF, the absorption is only 1.93 × 10^5^ m^−1^ and grows nonlinearly up to 8.07 × 10^5^ m^−1^ for $\alpha_{0}$ = 20 nm.

From the analysis of Figure 6, it can be inferred that ILF increases the absorption for QDDR structures with $h_{d}$ = 6 nm, 20 nm, but decreases it for $h_{d}$ = 8 nm. A clear blue-shift in the peaks appears for $h_{d}$ = 6 nm, while for $h_{d}$ = 8 nm, 20 nm, the peaks have an oscillatory behavior in energy.

**Figure 7:**
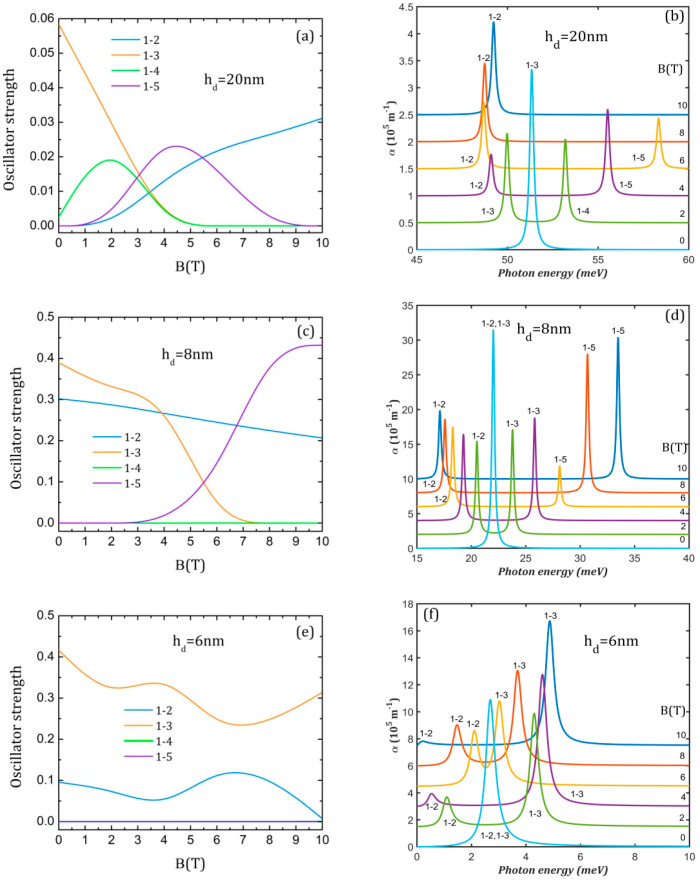
The oscillator strength (**a**,**c**,**e**) and the absorption spectra (**b**,**d**,**f**) at different values of the magnetic field B for the three QDDR structures considered. The transitions are indexed as 1–j, from the ground level to the excited j level. To avoid spectra overlapping, each spectrum is translated on the vertical axis.

Figure 7a,c,e present the oscillator strength behavior with the strengthening of the magnetic field. In this case, there are lower values than in Figure 6 due to the lower values taken by the transition moments since the magnetic field leads only to interchanges between the initial WFs and to a slight contraction.

For $h_{d}$ = 20 nm, we see in Figure 7a that the decrease in *O*_1–3_ is accompanied by an increase in *O*_1–2_. As can be inferred from Figure 4b, at B = 2 T, Φ_2_ is completely symmetric (*O*_1–2_ = 0), Φ_3_ is anti-symmetric to the *y*-axis (*O*_1–3_ ≠ 0) and Φ_4_ does not have a specific symmetry relative to the *y*-axis (*O*_1–4_ ≠ 0). Since Φ_1_ extends only on the dot while Φ_5_ covers only the rings, *O*_1–5_ = 0. At B = 4 T, (Φ_2_, Φ_3_) and also (Φ_4_, Φ_5_) have already exchanged their symmetry, so *O*_1–2_ ≠ 0, *O*_1–3_ = 0, *O*_1–4_ = 0 and *O*_1–5_ ≠ 0. At higher B, only *O*_1–2_ ≠ 0 since Φ_4_ becomes completely symmetric and Φ_3_ and Φ_5_ extend only on the rings. For $h_{d}$ = 8 nm (Figure 7c), *O*_1–2_ ≠ 0 for all B values since Φ_1_ and Φ_2_ do not change their symmetry, but it decreases due to WFs contraction. Up to B = 6 T, *O*_1–3_ ≠ 0 since Φ_3_ does not have a specific symmetry relative to the *y*-axis while *O*_1–4_ = 0 and *O*_1–5_ = 0 because their WFs are symmetric to the *y*-axis. At the further increment of the magnetic field, (Φ_3_, Φ_5_) exchange their symmetry so *O*_1–3_ = 0 and *O*_1–4_ = 0 but *O*_1–5_ ≠ 0. For $h_{d}$ = 6 nm (Figure 7e), *O*_1–2_ and *O*_1–3_ are non-zero for all B values but have different behaviors. *O*_1–2_ (*O*_1–3_) has a minimum (maximum) around B = 3.5 T related to symmetry exchange between Φ_1_ and Φ_2_ (Figure 4f, first row) and a maximum (minimum) around 6.5 T related to symmetry exchange between Φ_2_ and Φ_3_ (Figure 4f, middle row).

**Figure 8:**
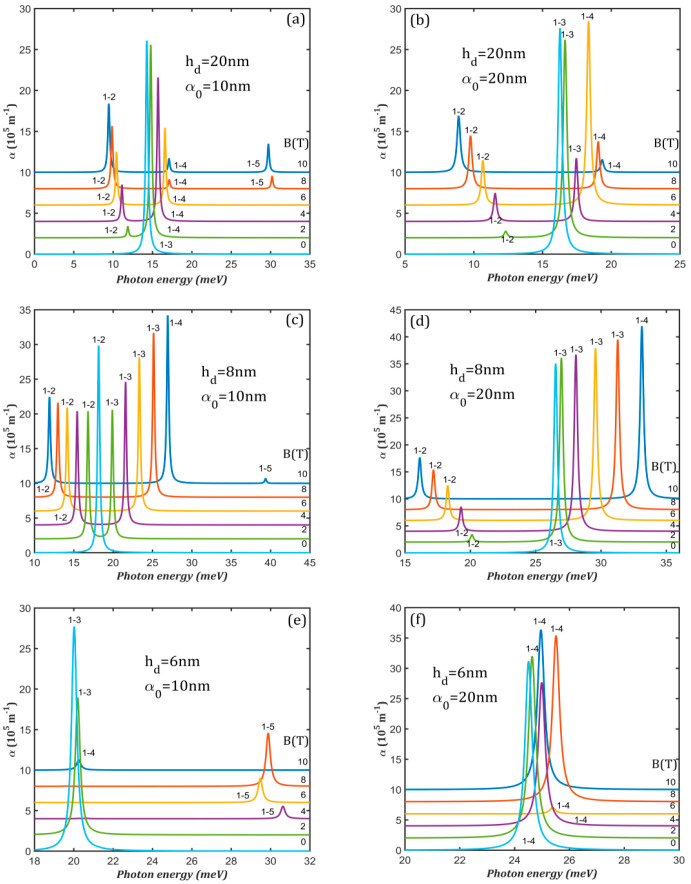
The absorption spectra at different values of the magnetic field B in the presence of the intense laser: (**a**) *h_d_* = 20 nm, α_0_ = 10 nm; (**b**) *h_d_* = 20 nm, α_0_ = 20 nm; (**c**) *h_d_* = 8 nm, α_0_ = 10 nm; (**d**) *h_d_* = 8 nm, α_0_ = 20 nm; (**e**) *h_d_* = 6 nm, α_0_ = 10 nm; (**f**) *h_d_* = 6 nm, α_0_ = 20 nm. The transitions are indexed as 1–j, from the ground level to the excited j level. To avoid spectra overlapping, each spectrum is translated on the vertical axis.

The magneto-optical absorption spectra represented in Figure 7 and Figure 8 are calculated for a probe laser of irradiation of 10^6^ W/m^2^. It can be noticed that the spectra from Figure 7 consist generally of two peaks in the presence of the magnetic field.

For $h_{d}$ = 20 nm (Figure 7b), the single 1→3 peak appearing for B = 0 at 51.34 meV is replaced by two peaks with oscillatory amplitudes at the increment of the magnetic field, one that is red-shifted up to 48.75 meV at B = 8 T and another that is blue-shifted up to 58.34 meV at B = 6 T. At higher B values, the spectrum shows again a single maximum corresponding to the 1→2 transition since only *O*_1–2_ ≠ 0. One can conclude that the presence of the magnetic field decreases the absorption maxima compared to its absence.

This is the same for $h_{d}$ = 8 nm (Figure 7d), where the maximum absorption is also obtained without the magnetic field, with the peaks 1→2 and 1→3 being superposed at 22.02 meV. The magnetic field separates the two peaks, with 1→2 being pushed toward lower energies and 1→3 to higher energies, being replaced at 6 T by a 1→5 peak. The splitting of the initial peak is 16.36 meV at B = 10 T, larger than any splitting obtained for $h_{d}$ = 20 nm.

For $h_{d}$ = 6 nm (Figure 7f), the initial large (1→2, 1→3) peak for B = 0 at 2.69 meV is also split in 1→2 and 1→3 peaks of oscillatory amplitude and energy. The maximum separation is only 4.86 meV, lower than the splitting obtained for the QDDR with $h_{d}$ = 20 nm and 8 nm.

The analysis of Figure 7 reveals that the magnetic field decreases the absorption and leads to spectra with two peaks of lower amplitude, with a separation that increases with the strengthening of the magnetic field. The maximum peaks displacement is obtained for the QDDR with $h_{d}$ = 20 nm and the minimum for the QDDR with $h_{d}$ = 6 nm.

Under the combined action of the laser and magnetic fields, the magneto-optical absorption has different characteristics. The magnetic field reduces again the absorption amplitude.

For $h_{d}$ = 20 nm at $\alpha_{0}$ = 10 nm (Figure 8a), the initial high 1→3 absorption maximum (for B = 0) is replaced by 1→2 and 1→4 peaks of lower amplitudes: 1→2 is red-shifted and its amplitude rises, while 1→4 is blue-shifted and its amplitude is lowered with increasing magnetic field. The maximum splitting between 1→2 and 1→4 absorption maxima is 7.65 meV at 10 T. A third peak, 1→5, appears for B = 8 T and 10 T. For $h_{d}$ = 20 nm at $\alpha_{0}$ = 20 nm (Figure 8b), the initial 1→3 peak is replaced by 1→2 and 1→3 peaks of lower amplitudes up to B = 6 T than by 1→2 and 1→4 due to the anti-crossing between E_3_ and E_4_ at 6 T (see Figure 5b). The largest splitting is 10.44 meV at 10 T due to a better separation of the energy levels.

For $h_{d}$ = 8 nm at $\alpha_{0}$ = 10 nm (Figure 8c), the magnetic field causes the apparition of a second 1→3 peak up to 8 T, accompanying the initial one corresponding to a 1→2 transition. At B = 10 T, the peak 1→3 is replaced by 1→4 due to the anti-crossing between E_3_ and E_4_ at 8.5 T (see Figure 5c). The peak 1→2 goes to red and lowers its intensity while 1→3 goes to blue and has increasing amplitude. The shift between 1→2 and 1→4 peaks at 10 T is 15.03 meV. For $h_{d}$ = 8 nm at $\alpha_{0}$ = 20 nm (Figure 8d), at B = 0, there is only 1→3 absorption maximum but at higher B values, a second 1→2 peak appears. Therefore, the spectra consist of two absorption maxima of increasing separation and amplitude, with the maximum value being 17.00 meV at 10 T.

For $h_{d}$ = 6 nm at $\alpha_{0}$ = 10 nm (Figure 8e), the spectra show a single absorption maximum that corresponds to a 1→3 transition for B < 4 T but to a 1→5 transition for B = 4-9 T. This is expected since Φ_5_ is *y*-axis-anti-symmetric on this domain due to the anti-crossing between E_5_ and E_6_ at 4 T (Figure 5e). After (E_5_, E_6_) anti-crossing at 9 T, Φ_5_ becomes symmetric, so the single peak corresponds to a 1→4 transition at B = 10 T, since Φ_4_ does not have a specific symmetry relative to the *x*- and *y*-axes. The spectra for $h_{d}$ = 6 nm at $\alpha_{0}$ = 20 nm (Figure 8f) consist of a singular 1→4 absorption maximum that is slightly blue-shifted up to B = 8 T, then slightly red-shifted (less than 1 meV). This is in agreement with the absence of anti-crossings in the energy levels considered in the simulation of the absorption spectra (Figure 5f).

The analysis of Figure 8 reveals that in the presence of ILF, the magnetic field decreases absorption and leads to spectra with two or even three peaks of lower amplitude, with a separation that increases both with B and $\alpha_{0}$ for QDDR with $h_{d}$ = 8 nm and 20 nm. For QDDR with $h_{d}$ = 6 nm, the magneto-optical absorption spectra are much simpler, consisting of a single peak of variable energy and amplitude.

## 4. Conclusions

We studied the electronic properties and the nonlinear absorption of GaAs/AlGaAs quantum dot–double quantum ring under the single and combined action of intense laser and magnetic fields. We performed full 3D numerical computations in the effective mass approximation. We analyzed three representative geometries that have different dot height while keeping the ring dimensions unchanged.

We showed that the intense THz laser field strongly modifies the confinement potential of QDDR and determines transitions from a dot–double-ring potential to dot–triple-ring or –multiple-ring potentials for a single material sample. It also determines the growth of all energies, generating a structure with multiple anti-crossings in the conduction band. On the contrary, the magnetic field leads to an oscillating multiple-crossing structure in the conduction band originated from the Aharonov–Bohm oscillations. Due to the closeness of the rings, we only observe the oscillations characteristic of a single large effective ring that covers more or less both rings depending on the dot height. In the presence of both laser and magnetic fields, some oscillations, not clearly defined, appear only for the upper levels. The electronic spectra only present anti-crossings and are displaced towards higher energies as the laser parameter is increased.

The intense laser field increases the absorption for the QDDR with small and large dot height but decreases it for medium dot height. The peaks display an oscillatory behavior in energy for large and medium dot height and a clear blue-shift for the structure with small dot height. The magnetic field generally decreases the absorption of QDDR. It leads to optical spectra with two peaks, one that is red-shifted and another that is blue-shifted for large and medium dot height. For the structure with small dot height, the peaks display oscillatory amplitude and energy. Under the combined action of both fields, the magneto-optical absorption spectra show the superposition of some of the individual effects of the two fields.

Our study reveals that the optical responses of QDDR to different fields are strongly influenced by the central dot height. The external fields efficiently control the absorption, enhancing or lowering the peaks and their separation, or displacing them to different energies, properties that are very useful in designing devices based on dot–double ring nanostructures.

## Data Availability

The datasets generated during and/or analyzed during the current study are available from the corresponding author on reasonable request.
